# Are acupoints specific for diseases? A systematic review of the randomized controlled trials with sham acupuncture controls

**DOI:** 10.1186/1749-8546-5-1

**Published:** 2010-01-12

**Authors:** Hongwei Zhang, Zhaoxiang Bian, Zhixiu Lin

**Affiliations:** 1 School of Chinese Medicine, Faculty of Science, The Chinese University of Hong Kong, Shatin, Hong Kong SAR, China; 2 School of Chinese Medicine, Hong Kong Baptist University, Kowloon Tong, Hong Kong SAR, China

## Abstract

**Background:**

The results of many clinical trials and experimental studies regarding acupoint specificity are contradictory. This review aims to investigate whether a difference in efficacy exists between ordinary acupuncture on specific acupoints and sham acupuncture controls on non-acupoints or on irrelevant acupoints.

**Methods:**

Databases including Medline, Embase, AMED and Chinese Biomedical Database were searched to identify randomized controlled trials published between 1998 and 2009 that compared traditional body acupuncture on acupoints with sham acupuncture controls on irrelevant acupoints or non-acupoints with the same needling depth. The Cochrane Collaboration's tool for assessing risk of bias was employed to address the quality of the included trials.

**Results:**

Twelve acupuncture clinical trials with sham acupuncture controls were identified and included in the review. The conditions treated varied. Half of the included trials had positive results on the primary outcomes and demonstrated acupoint specificity. However, among those six trials (total sample size: 985) with low risk of bias, five trials (sample size: 940) showed no statistically significant difference between proper and sham acupuncture treatments.

**Conclusion:**

This review did not demonstrate the existence of acupoint specificity. Further clinical trials with larger sample sizes, optimal acupuncture treatment protocols and appropriate sham acupuncture controls are required to resolve this important issue.

## Background

In acupuncture, the acupoints for a specific treatment are selected from a group consisting of local acupoints, distal acupoints and symptomatic acupoints. The selection should be in accordance with the meridian principles and the characteristics of acupoints. However, it was claimed that acupuncture may be effective even when the needle is inserted anywhere in the appropriate segment or at motor points [1,2] for some disorders such as nausea but not others such as chronic pain [3,4]. Although acupuncture treatment may regulate physiological functions [5], the current understanding of its mechanisms in physiological and psychosocial aspects is inadequate to explain the effects of specific acupoints [6-8]. There have been many clinical trials and experimental studies on the specificity of acupoints [3,9,10] but systematic reviews are not available to show any clear picture of the current evidence.

The use of controlled needling in clinical trials of acupuncture has varied considerably [11,12]. The three most commonly used controlled needling methods are sham acupuncture (on points away from treatment acupoints), minimal acupuncture (superficial needling) and placebo acupuncture (noninvasive needling). The treatment effect produced by acupuncture may be attributed to three main components: (1) a nonspecific placebo effect, which is related to patients' expectation and the interaction between patients and acupuncturists; (2) a general physiological effect due to needles being inserted into the skin; and (3) the specific effect due to needling manipulation at the specific acupoints [13]. To examine whether an efficacy difference between traditional acupuncture on specific acupoints and sham acupuncture controls at sites away from conventional acupoints, we conducted a systematic review of randomized controlled trials using sham acupuncture controls published between 1998 and 2009. Although there are different definitions of sham acupuncture controls [14], in this article, sham acupuncture is considered as needling at sites away from conventional acupoints with the same needling depth and stimulation procedures as those of conventional acupuncture.

## Methods

### Search strategy

We searched the databases Medline, Embase, AMED, and Chinese Biomedical Database (CBM) in March 2009. The search strategy for the English language databases was an "OR" combination of the terms "sham acupuncture", "sham needle", "placebo acupuncture" and "placebo needle". The search results were then limited to the reports of randomized controlled trials published between1998 and 2009. Slight syntax modifications to the search strategy were made to suit various English language databases. In the Chinese language CBM, the search strategy used an "OR" combination of the terms *jiazhen *(sham acupuncture), *anweizhen *(placebo acupuncture) and *feixueweizhen *(non-acupoint acupuncture) and the search result was limited to the reports of human studies published between 1998 and 2009.

### Inclusion criteria

The randomized controlled trials evaluating the effectiveness or efficacy of main body acupuncture treatment with sham acupuncture as a control were included. The conventional acupuncture treatment was on the conventional acupoints with manual manipulation according to Chinese medicine theory. Sham acupuncture was applied at sites away from the conventional acupoints while having the same needling depth and stimulation procedures.

### Exclusion criteria

The studies involving scalp acupuncture, electro-acupuncture, tongue acupuncture, auricular acupuncture, abdominal acupuncture, laser acupuncture, intradermal needles, acupoint injection and trials on healthy subjects were excluded.

### Data extraction

One author (HWZ) extracted data which were then verified by the other two authors. For each included study, we collected information about the study design, sample size, treated clinical problem, pattern of acupuncture treatment, professional experience of the acupuncturists, characteristics of proper and sham acupuncture treatment procedures (such as treatment sites, *deqi *sensation, needle retention time and number of treatment sessions and frequency) and primary outcome.

### Risk of bias assessment

The Cochrane Collaboration's tool for assessing risk of bias was used to evaluate the risk of bias of the following key aspects: sequence generation; allocation concealment; blinding of participants, personnel and outcome assessors; and incomplete outcome data [15]. The risk of bias for the main outcomes within and across studies was evaluated as follows: (1) low risk of bias, which is unlikely to alter the results significantly; (2) unclear risk of bias, which raises some doubt about the results; and (3) high risk of bias, which seriously weakens the confidence in the results. When all key aspects within a trial were classified as low risk of bias or most information was obtained from trials at low risk of bias, the risk of bias of the outcome was classified as low. When all key aspects were classified as low or unclear risk of bias or most information was obtained from trials at low or unclear risk of bias, the risk of bias of the outcome was classified as unclear. Likewise, when one or more key aspects were classified as high risk of bias or the proportion of information from trials at high risk of bias was sufficient to affect the interpretation of results, the risk of bias for the outcome across trails was classified as high [15].

### Data analysis

The trial data were tabulated and then qualitatively analyzed to determine the risk of bias, trial characteristics and proper and sham acupuncture treatments. Quantitative synthesis was not conducted.

## Results

### Search results

**Figure 1:**
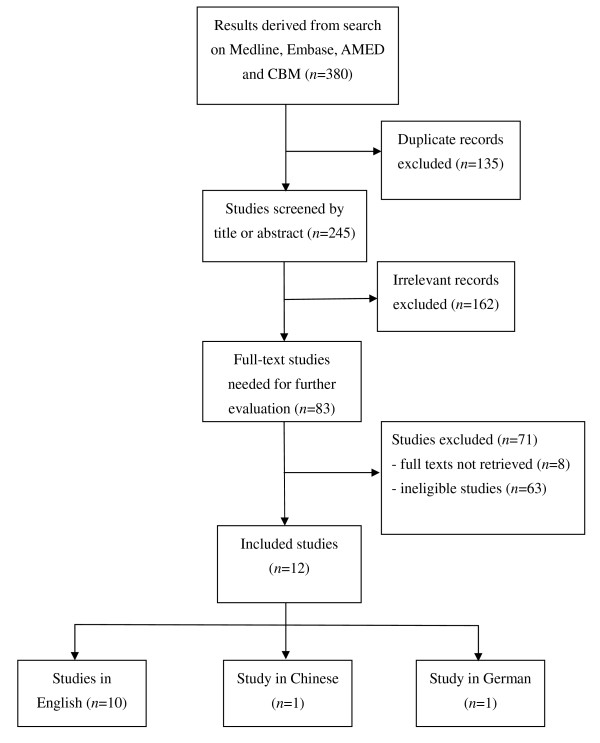
**Flow chart showing the retrieval process of clinical trial reports included in the systematic review**.

The initial search generated a total of 380 articles from multiple databases, of which 245 articles were retained for screening after duplicates were removed (Figure 1). We screened the titles and abstracts of these articles and identified 83 eligible articles whose full texts were needed to retrieve for further evaluation. The full texts of 74 articles were available. Twelve articles, of which ten were in English [16-25], one in Chinese [26] and one in German [27], were included for qualitative analysis. Although the full text of the German language article was not available, its eligibility for inclusion was ensured according to the information in the abstract. The other trials were excluded mainly due to the following reasons: use of minimal sham acupuncture or noninvasive placebo acupuncture as controls; acupuncture treatment combined with electronic stimulation or other treatment approaches, such as acupoint massage or scalp acupuncture; and no random allocation.

### Trial quality

Of the 12 included trials, six (50%) had a low risk of bias, while five trails (41.7%) had an unclear risk of bias, and one trial had a high risk of bias (Table 1). The main problems related to the trial quality include poor description of the sequence generation and allocation concealment methods and insufficient reporting or not addressing missing data of outcomes.

**Table 1:** Trial quality of randomized controlled trials with sham acupuncture control

Trial ID	Risk of bias	Sample size/based on calculation^a^	Primary outcome	Result^b^
Huang 2008 [26]	Unclear	120/No	Global symptoms	+
Flachskampf 2007 [17]	Unclear	160/Yes	Average systolic and diastolic blood pressure	+
Vincent 2007 [21]	Low	103/Yes	Hot flash scores	-
Assefi 2005 [22]^c ^	Low	100/Yes	Pain (VAS scores)	-
Emmons 2005 [23]	Low	85/Yes	Number of incontinent episodes	-
Forbes 2005 [20]	Low	59/Yes	Self-rated symptom scores	-
Karst 2004 [27]	Unclear	54/unkown	Pain intensity	+
Fink 2002 [18]	Low	45/Yes	Pain (VAS scores)	+
Smith 2002 [24]	Low	593/Yes	Nausea (self-rated)	-
Fireman 2001 [19]	High	32/No	Overall symptoms (VAS scores)	+
Wang 2000 [25]	Unclear	132/No	Pain (VAS scores)	+
Biernacki 1998 [16]	Unclear	23/No	Spirometric value	-

^a ^Sample size calculation based on the power analysis intended to detect the difference between proper and sham acupuncture treatment.

^b^"+" means that the trial detected different outcomes between proper and sham acupuncture; "-" denotes that a trial did not detect different outcomes between proper and sham acupuncture.

^c ^The proper acupuncture treatment was compared to the pooled sham acupuncture groups (including acupuncture for an unrelated condition, needle insertion at non-acupoints, or noninsertive simulated acupuncture).

Due to the difficulty in blinding the acupuncturists in clinical trials, most trials blinded the patients or outcome assessors. Only three included trials assessed the degree of blinding by asking the participants to guess whether the treatment was sham acupuncture. Two of the three trials showed no significant difference between the proper and sham acupuncture groups in terms of the proportion of participants who thought they received proper acupuncture; and these two trials showed no significant difference in the main outcome between the proper and sham acupuncture groups [21,22]. The other trial showed a significant difference in the proportion of participants who thought they had received proper acupuncture, indicating unsuccessful blinding of the sham acupuncture. This trial also reported a significant beneficial effect on the traditional acupuncture group [24,28].

Seven out of the 12 included trials determined sample sizes through power analysis [17,18,20-24]. Another four trials had sample sizes from 23 to132 [16,19,25,26]. The last trial [27] had a sample size of 54 without providing information about sample size calculation in the abstract.

### Proper acupuncture treatment

All the acupuncture treatments in the randomized controlled trials were based on traditional Chinese acupuncture principles. Six out of the 12 trials had a standardized treatment protocol with the same acupoints for all patients [16,18,19,21,23,26], whereas three trials used individualized treatment with various acupoints based on the syndrome differentiations of the patients [17,20,24]. Two trials used a half-standardized treatment protocol in which acupoints were selected from a group of acupoints according to traditional Chinese acupuncture principles [22,25].

Most trials (75%) did not mention the professional experience of the acupuncturists. The number of acupoints used in the proper acupuncture groups ranged from one to 16. Most trials used six to eight acupoints for one treatment. *Deqi *sensation was reported in half of the 12 trials. The needle retention time was about 15-30 minutes. The numbers of treatment sessions varied from one to 24, and the treatment frequencies were from one to six times per week. Eight trials chose a treatment frequency of less than twice a week [16,19-24,27], whereas three studies treated patients more than twice a week [17,25,26]. No acupuncture treatment-related information was reported in the trial by Fink *et al*. [18]. Additional file 1 summarizes the treatment characteristics of the randomized controlled trials with sham acupuncture controls included in this review.

### Sham acupuncture control

Three approaches were employed to choose the sham treatment sites. Of the 12 trials, nine chose non-acupoints, which may lie in nearby areas, generally 2 cm or 5 cm away from the proper treatment acupoints, or far away on the body [16,18,20-22,24-27]. Three trials chose acupoints that were purportedly good for other unrelated conditions. For example, one trial chose acupoints for relaxation when treating patients with overactive bladder with urge incontinence [23], while another trial chose BL-60 (*Kunlun*) when treating patients with irritable bowel syndrome [19]. The trial on patients with fibromyalgia had both kinds of control methods, with one using acupoints intended for treatment of early menses and one using non-acupoints as the treatment sites [22]. One study chose non-acupoints on the same meridian as the sham treatment sites [17].

In the included trials, the treatment procedures and needling manipulation of sham acupuncture were not described in detail. In five trials, the investigators only reported that same treatment techniques and procedures were applied in the sham control groups, without specifying the techniques and procedures actually used [17,18,20,23,26]. There was no mention of the needling manipulation of sham acupuncture in other trials [16,19-22,24,25]. Only two reports specified the same standard needling depths for the two groups [22,27]. Two trials reported that no *deqi *sensation was experienced by the sham acupuncture groups [20,25].

### Treated conditions and outcome measures

The treated conditions in all the included trials involved chronic disorders, including ischemic stroke, hypertension, hot flashes, irritable bowel syndrome, fibromyalgia, overactive bladder with urge incontinence, chronic epicondylitis, nausea or vomiting during early pregnancy and stable asthma. One trial enrolled patients suffering from both chronic and acute low back pain [25]. Four trials involved pain-related problems [18,22,25,27]. The trial conducted in China enrolled only the patients suffering from ischemic stroke with blood stagnation in collaterals (*luomai*) [26].

Ten trials employed subjective primary outcomes assessed by patients themselves or data collectors [18-27]. Only two trials employed objective measures for assessing primary outcomes, namely blood pressure and spirometric value respectively [16,17].

### Trial results

Among the twelve included trials, six [17-19,25-27] produced positive results favoring proper acupuncture treatment on the primary outcomes and the remaining six had negative results showing no significant difference between proper and sham acupuncture treatments. Among the six trials with low risk of bias, five (83.3%) showed negative results. Conversely, five out of the six trials [17,19,25-27] with unclear or high risk of bias showed positive results (Tables 1 and Additional file 1).

Of the seven trials that used sample size calculation, five [20-24] (71.4%) produced negative results. Among the four trials that did not report sample size calculation, only one [16] (25%) produced a negative result.

Of the six studies using conventional acupuncture treatment (i.e. same acupoints for all participants), three [18,19,26] produced positive results. Similar results were found in the two trials [22,25] with semi-conventional acupuncture treatment. Among the three trials with individualized acupuncture treatment, only one trial [17] produced positive results.

Among the eight trials with a treatment frequency of only once or twice a week, two [25,27] trials showed positive results. The three trials [17,19,26] with more frequent treatments had positive results.

Among the eight trials with non-acupoints as the sham treatment sites, four had positive results [16,18,20,21,24-27]. Of the three trials using acupoints for unrelated condition or non-acupoints on the same meridian as the sham treatment, two had negative results [17,19,23]. The remaining trial that pooled the results of sham acupuncture control groups (including acupuncture for unrelated conditions, needle insertion at non-acupoint locations and noninsertive acupuncture) showed negative results [22].

Among the four trials of pain-related problems [18,22,25,27], only one trial generated negative results [25]. Two trials conducted by the same research team on chronic epicondylitis showed a significant difference between the proper and sham acupuncture groups [18,27]. The results of the two trials on irritable bowel syndrome were divergent [19,20].

## Discussion

The present study systematically reviewed the randomized controlled trials of acupuncture employing sham acupuncture as controls published between 1998 and 2009. Evidence for the specificity of acupoints is heterogeneous, and no definitive conclusion could be drawn. We found that positive results suggesting the existence of acupoint specificity were more often seen in the trials with low quality, insufficient sample sizes and high acupuncture treatment frequency. No association was established between the trial results and the pattern of acupuncture treatment (standardized or individualized), the selection of treatment sites in the sham acupuncture group, the kind of disorders, or the outcome measures employed (objective or subjective).

### Trial quality

We could not exclude the possibility that the low quality of the trials may have resulted in an overestimate of the trial outcomes. Trials with inadequate random allocation, poor blinding and missing outcome data after randomization tend to overestimate the results [29,30]. The generally low quality of the trials with small sample size may explain why more positive results were found in these trials.

### Proper acupuncture treatment and sham control

There has been no consensus on how to determine the optimal acupuncture treatment whose efficacy is affected by the selection of acupoints, needling depth, manipulation techniques, treatment frequency and total number of treatment sessions [13,31]. The acupuncturist's professional ability is also an important factor. In the included trials, information about the acupuncture treatment procedures and acupuncturist's professional experience were insufficient. In one trial, for example, the chosen treatment frequency was based on practical feasibility rather than rational consideration of effectiveness [24]. A significant difference was demonstrated between proper and sham acupuncture when both groups received reinforcing needling techniques, suggesting that proper acupoints are more susceptible to needling manipulation [31]. It is possible that insufficient needling stimulation partially contributed to the negative trial results showing no acupoint specificity. The discrepancies in the pattern of acupuncture treatment and needling stimulation may explain the contradictory results from the two trials on irritable bowel syndrome [19,20].

According to Chinese medicine principles, acupoint selection based on syndrome differentiation is crucial for treatment effectiveness. All the included trials except one in China provided no information regarding the syndrome differentiation on the subjects. Apart from the claim that the trial used individualized treatment or treatment according to Chinese medicine, no further information and rationale on acupoint selection were provided in these trials.

The selection of acupoints, needling depth, manipulation techniques and the number and frequency of treatment sessions are important components of acupuncture treatment that may work together to achieve effectiveness. For studying acupoint specificity, these components of the sham acupuncture control should be identical to the proper acupuncture treatment except for the treatment sites. In the included trials, a detailed description about needling in the sham acupuncture was generally absent. In two trials [22,23], the sham needles were only inserted into the skin without further manipulation. The absence of needling manipulation of sham acupuncture, in contrast to proper acupuncture, may generate false positive trial results regarding acupoint specificity. The sites of sham acupuncture should also be selected carefully. Based on Chinese medicine theory, it is possible that the acupoints for other unrelated conditions or non-acupoints on the meridian can also exert a certain degree of therapeutic effects. Therefore, non-acupoints outside the channel of meridian may be a better choice for sham acupuncture when studying acupoint specificity.

### Conditions treated

Based on the current review, it seems that acupoints are specific for some disorders such as hypertension, but not specific for others such as fibromyalgia [22,32,33]. The peripheral and central sensitization in the patients with fibromyalgia syndrome may explain the nonspecificity of acupoints. However, due to insufficient evidence, the causal relationship between specific acupoints and treatment effects cannot be confirmed.

### Strengths and limitations

Due to resource limitations, we could only review the trials published after 1998. The publications on acupuncture trials during this period are believed to have better quality than those published earlier, largely owing to the availability of STRICTA and CONSORT guidelines [34]. Our findings on acupoint specificity are consistent with a previous review [11].

### Future research

A more thorough systematic review covering all available randomized controlled trials with sham acupuncture controls would be of great help in elucidating the acupoint specificity. Further reviewing on clinical acupuncture trials using minimal acupuncture and noninvasive needles with different needling depth and manipulation would also help resolve the issue of acupoint specificity. When developing clinical trials to study acupoint specificity, special attention should be given to the following four aspects: (1) random sequence generation and allocation concealment, blinding and the completeness of outcome measures should be addressed clearly; (2) adequate sample size is crucial to detect the difference between proper and sham acupuncture; (3) the treatment procedures including acupoint selection, needling depth and manipulation, number and frequency of treatment sessions, needle retention time and availability of *deqi *sensation should be optimized before actual clinical trials; (4) the treatment sites of sham acupuncture should be selected carefully, preferably the non-acupoints outside meridian channels. The treatment procedures of sham acupuncture should be as comparable as possible to those of proper acupuncture except for treatment sites. Minimal acupuncture should not be used as a sham control for studying acupoint specificity as it is not considered a valid placebo in randomized controlled trials of acupuncture [35].

## Conclusion

Acupoint specificity cannot be confirmed due to the paucity of available high-quality empirical evidence. Further clinical trials with sufficient sample sizes, optimal acupuncture treatment protocols and appropriate sham acupuncture controls are required to clarify this important issue.

## Competing interests

The authors declare that they have no competing interests.

## Authors' contributions

HWZ conceived the study, did the literature search, performed data extraction and drafted the manuscript. ZXL and ZXB verified the extracted data and assisted in the manuscript preparation. All authors read and approved the final version of the manuscript.

## Supplementary Material

Additional file 1 **Treatment characteristics of randomized controlled trials with sham acupuncture control**. This table summarizes the treatment characteristics of the randomized controlled trials with sham acupuncture controls included in this review. Click here for file
