# Supplementary material for: Are acupoints specific for diseases? A systematic review of the randomized controlled trials with sham acupuncture controls
Source: Chin Med. 2010 Jan 12;5:1. doi: 10.1186/1749-8546-5-1 (PMC2818640; doi:10.1186/1749-8546-5-1)
Supplement: Additional file 1 — Treatment characteristics of randomized controlled trials with sham acupuncture control. This table summarizes the treatment characteristics of the randomized controlled trials with sham acupuncture controls included in this review. [file 1749-8546-5-1-S1.DOC]

Treatment characteristics of randomized controlled trials with sham acupuncture control

| **Trial ID** | **Disorder** | **Standardized or individualized treatment** | **Acupuncturist**  **(years of experience)** | **Number of treatment sites** | ***Deqi*** | **Needle retention time (min)** | **Treatment sessions and frequency (or duration)** | **Treatment sites of sham acupuncture** |
| --- | --- | --- | --- | --- | --- | --- | --- | --- |
| Huang 2008 [26] | Ischemic stroke | Standardized | Unclear | 6–8 | Yes | 30 | 24, 6 per week | Nearby (0.5cm) non-acupoints |
| Flachskampf  2007 [17] | Hypertension | Individualized | Several | 5–6 | Unclear | 20 | 22, 3 or 5 per week | Non-acupoints on the meridian, |
| Vincent  2007 [21] | Hot flashes | Standardized | <1 | 12 | Yes | 30 | 10, 2 per week | Nearby (5cm or more away) non-acupoints |
| Assefi 2005 [22] | Fibromyalgia | Semi-standardized | 4–18 | <14 | Unclear | 30 | 24, 2 per week | Acupoints for unrelated condition;  non-acupoints |
| Emmons 2005 [23] | Overactive bladder  with urge incontinence | Standardized | Unclear | 7 | Yes | 20 | 4, 1 per week | Acupoints for unrelated condition |
| Forbes 2005 [20] | Irritable bowel syndrome | Individualized | Unclear | 8–16 | Yes | 25 | 10, 1 per week | Non-acupoints |
| Karst 2004 [27] | Chronic lateral epicondylitis | Unclear | Unclear | Unclear | Unclear | Unclear | 10, 2 per week | Non-acupoints |
| Fink 2002 [18] | Chronic epicondylitis | Standardized | Unclear | 6 | Unclear | 25 | Unclear | Nearby (5 cm or more away) non-acupoints |
| Smith 2002 [24] | Nausea or vomiting during early pregnancy | Individualized | Unclear | 3–6 | Yes | 20 | 5, 1 or 2 per week | Nearby non-acupoints |
| Fireman  2001 [19] ^a^ | Irritable bowel syndrome | Standardized | Unclear | 1 | Unclear | 30 | 2 (over 4 weeks) | Acupoints for unrelated condition |
| Wang 2000 [25] | Pain before and after lumbar disc protrusion surgery | Semi-standardized | Unclear | 5–9 | Yes | 15 | 2 or 3 (before and after surgery for a total of 3–6 days) | Nearby (2 cm away) non-acupoints |
| Biernacki 1998 [16] | Stable asthma | Standardized | Unclear | 1 | Unclear | 20 | 1 | Non-acupoints |

^a^ The result of the treatment before cross-over was used for assessment in this randomized cross-over study.
